# Conditionally reprogrammed asthmatic bronchial epithelial cells express lower *FOXJ1* at terminal differentiation and lower *IFNs* following RV-A1 infection

**DOI:** 10.1152/ajplung.00230.2022

**Published:** 2022-08-30

**Authors:** Punnam Chander Veerati, Kristy S. Nichol, Jane M. Read, Nathan W. Bartlett, Peter A. B. Wark, Darryl A. Knight, Christopher L. Grainge, Andrew T. Reid

**Affiliations:** ^1^School of Medicine and Public Health, University of Newcastle, Callaghan, New South Wales, Australia; ^2^Asthma and Breathing Research Program, Hunter Medical Research Institute, University of Newcastle, New Lambton Heights, New South Wales, Australia; ^3^Immune Health Program, Hunter Medical Research Institute, University of Newcastle, New Lambton Heights, New South Wales, Australia; ^4^School of Biomedical Sciences and Pharmacy, University of Newcastle, Callaghan, New South Wales, Australia; ^5^Department of Respiratory and Sleep Medicine, John Hunter Hospital, New Lambton Heights, New South Wales, Australia; ^6^Department of Anesthesiology, Pharmacology and Therapeutics, University of British Columbia, Vancouver, British Columbia, Canada; ^7^Research and Academic Affairs, Providence Health Care Research Institute, Vancouver, British Columbia, Canada

**Keywords:** asthma, conditional reprogramming (CR), FOXJ1, interferon, rhinovirus

## Abstract

Primary bronchial epithelial cells (pBECs) obtained from donors have limited proliferation capacity. Recently, conditional reprogramming (CR) technique has overcome this and has provided the potential for extended passaging and subsequent differentiation of cells at air-liquid interface (ALI). However, there has been no donor-specific comparison of cell morphology, baseline gene expression, barrier function, and antiviral responses compared with their “parent” pBECs, especially cells obtained from donors with asthma. We, therefore, collected and differentiated pBECs at ALI from mild donors with asthma (*n* = 6) for the parent group. The same cells were conditionally reprogrammed and later differentiated at ALI. Barrier function was measured during the differentiation phase. Morphology and baseline gene expression were compared at terminal differentiation. Viral replication kinetics and antiviral responses were assessed following rhinovirus (RV) infection over 96 h. Barrier function during the differentiation phase and cell structural morphology at terminal differentiation appear similar in both parent and CR groups, however, there were elongated cell structures superficial to basal cells and significantly lower *FOXJ1* expression in CR group. *IFN* gene expression was also significantly lower in CR group compared with parent asthma group following RV infection. The CR technique is a beneficial tool to proliferate pBECs over extended passages. Considering lower *FOXJ1* expression, viral replication kinetics and antiviral responses, a cautious approach should be taken while choosing CR cells for experiments. In addition, as lab-to-lab cell culture techniques vary, the most appropriate technique must be utilized to best match individual cell functions and morphologies to address specific research questions and experimental reproducibility across the labs.

## Graphical abstract

**Figure:**
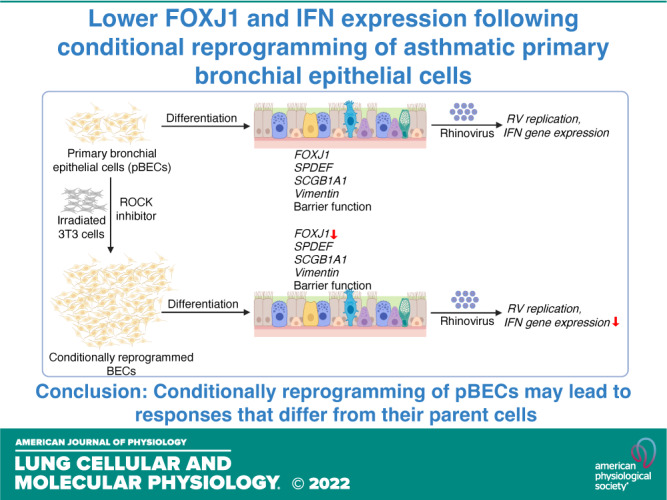

## INTRODUCTION

Recent advancements in cell culture have now made it possible to proliferate primary bronchial epithelial cells (pBECs) ad infinitum, greatly amplifying cell number without any genetic modifications in an otherwise growth-restricted population (1, 2). This tool, termed conditional reprogramming, has enabled the more extensive use of pBECs derived from healthy donors as well as those with respiratory diseases, such as cystic fibrosis and asthma, where large quantities of primary cells are difficult to obtain (3–5). For example, using this technique, cystic fibrosis pBECs were grown for extended passages and were able to be differentiated at air-liquid interface (ALI) culture (3). However, with an increase in passage numbers, there were fewer multiciliated cells at terminal differentiation and a decline in CFTR function, which was restored by creating a hypoxic environment (2% oxygen) during the expansion phase (3, 6). Asthma-derived cells were also conditionally reprogrammed (CR) and reported to differentiate at ALI culture from passage one CR cells (4). However, comparison of CR cells morphology, baseline gene expression and barrier function with their “parent” asthma pBECs at terminal differentiation were not investigated.

Patients with asthma experience virus-induced exacerbations, particularly caused by rhinovirus (RV), with impaired or delayed antiviral innate immune responses from pBECs (7–13). There is limited literature on how differentiated CR cells respond to viral infections (14, 15). In previous work, CR cells from healthy donors and IL-13-treated CR cells were infected with a virus or viral mimic; however, reports of these studies lack a detailed examination of antiviral interferon responses (14, 15). There was an induction of interferon-λ1 (IFN-λ1) protein from differentiated CR cells from infant donors following exposure to double-stranded (ds) RNA [polyinosinic:polycytidylic acid (Poly (I:C)] (14). Exposure to RV-16, a major group RV, induced secretion of interferon γ-induced protein-10 (IP-10) from IL-13-treated CR cells (to mimic asthma) at an early phase of ALI differentiation (*day 14*) (15). But this study did not report antiviral innate immune responses such as IFN-β and IFN-λs following RV-16 infection (15). There is a need to better understand and identify how differentiated CR asthma cells respond to viral infections before using them for physiological or interventional studies.

To date, no direct comparison of antiviral innate immune responses, inflammatory responses, and viral replication kinetics has been made between differentiated asthma-derived pBECs and matched differentiated CR cells from the same donor. At the same time, cell morphology, barrier function, and baseline gene expression of donor-matched CR cells have not been compared. Therefore, we hypothesize that CR asthma-derived cells will differentiate at ALI culture and respond to viral infections similar to their “parent” asthma-derived pBECs. Here, we aim to investigate the host epithelial innate immune responses to RV infection and also evaluate the morphology of differentiated CR asthma cells to their parent pBECs.

## MATERIALS AND METHODS

### Ethics, Primary Bronchial Epithelial Cells (pBECs) Collection, Expansion, and Differentiation at Air-Liquid Interface (ALI) Culture

All experiments were conducted in accordance with Hunter New England Area Health Service Ethics Committee (05/08/10/3.09) and the University of Newcastle Safety Committee (H-163-1205) approvals. All pBECs were obtained from bronchial brushing during fiber-optic bronchoscopy from individuals with prior written informed consent. These donors were clinically assessed as asthmatic and further classified as mild persistent disease, as defined by the Global Initiative for Asthma (GINA; 12, 16). All donors were nonsmokers during the cell collection and their clinical characteristics are shown in Table 1. Cells were maintained in Bronchial Epithelial Cell Growth Media (BEGM; Lonza) for expansion, when confluent seeded at 2 × 10^5^ cells/cm^2^ on 12 mm diameter transwell inserts with 0.4 μm pore size (Corning) as previously described (13). Cells were exposed to air when confluent on insert for differentiation with basal media changes every 2–3 days. Transepithelial electrical resistance (TEER) measurements were made every 7 days at ALI using an epithelial volt-ohm meter (EVOM2; World Precision Instruments).

**Table 1.** Clinical characteristics of donors with asthma used in this study

Characteristic	Value
Number, *n*	6
Severity (*n*)	Mild (6)
Smoking history (former/never)	2/4
Age, yr (SD)	50.8 (17)
Male, *n* (%)	4 (66.7)
FEV1, % predicted (SD)	83.7 (6.2)
FVC, % predicted (SD)	83.1 (6.9)
(FEV1/FVC) % (SD)	77.3 (4.3)
BAL cell count (E/N/P)	2/1/3
Atopy (SPT positive)	2
ICS dose, BDP† equivalent, mg (SD)	400 (141)#

BAL, bronchoalveolar lavage; FEV1, forced expiratory volume in 1 s; FVC, forced vital capacity; ICS, inhaled corticosteroid; E, eosinophilic; N, neutrophilic; P, paucigranular; SPT, skin prick test. †ICS doses were adjusted to beclomethasone dipropionate (BDP) equivalent. #Three out of six donors were on ICS treatment.

### Conditionally Reprogramming Technique

The above pBECs from donors with asthma were used in combination with γ-irradiated NIH-3T3 fibroblasts and Rho-associated protein kinase (ROCK) inhibitor (Y-27632) to generate CR BEC populations as previously described (2, 4, 5). These cells were cocultured in a modified growth medium (CR medium) containing a 2:1 mix of Ham’s F12 and Dulbecco’s modified Eagle’s medium (DMEM) high glucose + l-glutamine (Sigma-Aldrich) with supplements. Upon 50% cell confluency, CR media was gradually weaned with BEGM to remove the ROCK inhibitor during expansion phase. At 50% confluency, media was switched to 70:30 (CR media: BEGM), at 70% confluency to 50:50, and at 85% confluency to 50(no ROCKi):50. A differential trypsinization protocol was followed to separate irradiated fibroblasts (NIH-3T3) and CR-BECs. Once isolated, CR-BECs were seeded on transwells and cultured as per pBEC ALI protocol.

### Rhinovirus (RV) Infection and Sampling

Following cell differentiation, cells were incubated with human rhinovirus A1 (RV-A1) at multiplicity of infection (MOI) 0.001 or media control for 6 h as previously described (13). Following infection, cells were harvested for apical media, basolateral media, RNA using RLT buffer, and cells fixed with formalin at 0, 24, 48, 72, and 96 h postinfection (hpi).

### Alcian Blue/Periodic Acid Schiff Staining

ALI membranes were coated apically with 2% wt/vol low melting point agarose (Sigma-Aldrich) and were subjected to standard histological ethanol/xylene dehydration followed by paraffin embedding as previously described (17). Standard alcian blue pH 2.5 and periodic acid Schiff staining (AB/PAS) were performed on 5 µm thick ALI sections to observe total mucin content as previously described (17, 18).

### Immunofluorescent Labeling

Sectioned ALI membranes were subjected to immunofluorescent labeling as previously described (17). Primary antibodies; anti-acetylated tubulin (T7451; Sigma-Aldrich), anti-smooth muscle actin (ab32575; Abcam, UK), anti-MUC5AC (ab3649; Abcam, UK), and anti-P63 (ab124762; Abcam, UK) were incubated overnight at 4°C followed by an appropriate goat anti-mouse 594 (ab150116; Abcam, UK) or goat anti-rabbit 488 secondary antibodies (4412S; Cell-Signaling Technology). Cell nuclei were counterstained with DAPI [2-(4-aminophenyl)-1H -indole-6-carboxamidine] (19).

### Gene Expression Analysis Using Quantitative PCR (qPCR)

Total RNA was extracted from cells lysed in RLT buffer during harvest by using the RNeasy mini kit (Qiagen, Netherlands), and concentration was determined by using a Nanodrop spectrophotometer (Thermo Fisher Scientific). For gene expression analysis, the total RNA (200 ng) was reverse transcribed into cDNA using high-capacity cDNA reverse transcription kit (Thermo Fisher Scientific). Absolute quantification was performed with customized specific primers and probes for viral RNA and IFN genes and normalized to 18S rRNA as previously described (20). Relative quantification was performed with TaqMan gene expression assays (Themo Fisher Scientific) for baseline gene expression of *FOXJ1* (Hs00201755_m1), *SPDEF* (Hs0017942_m1), *SCGB1A1* (Hs00171092_m1), and *Vimentin* (Hs00418522_m1) on an ABI 7500 fast real-time PCR system (Applied Biosystems).

### Extracellular Protein Analysis Using LEGENDplex

Apical supernatant from cells at each harvest time point was assessed using LEGENDPlex human antivirus response panel (BioLegend), as per the manufacturer’s instructions. Antiviral interferons (IFN-β, λ1, and λ2/3) and inflammatory cytokines including interleukins (IL-1β, 6), tumor necrosis factor-α (TNF-α), interferon-γ inducible protein (CXCL10/IP-10), and granulocyte-macrophage colony-stimulating factor (GM-CSF) were measured.

### Statistical Analysis

All statistical analyses were performed with GraphPad Prism 9.00 software (La Jolla, CA). Normality was tested with the D’Agostino and Pearson test and the Anderson–Darling test. Non-normally distributed data were represented as median (interquartile range, IQR). Statistical analysis of multiple groups at a given time point was performed using a nonparametric Kruskal–Wallis test with Dunn’s multiple corrections. Statistical analysis for two groups was performed using a two-tailed Wilcoxon matched-pairs signed rank test. *P* values < 0.05 were considered statistically significant.

## RESULTS

### Differentiated CR Cells Are Morphologically Similar to Their Parent Asthma Cells at ALI Culture except with an Elongated Cytoskeleton Superficial to Basal Cells

**Figure 1.**
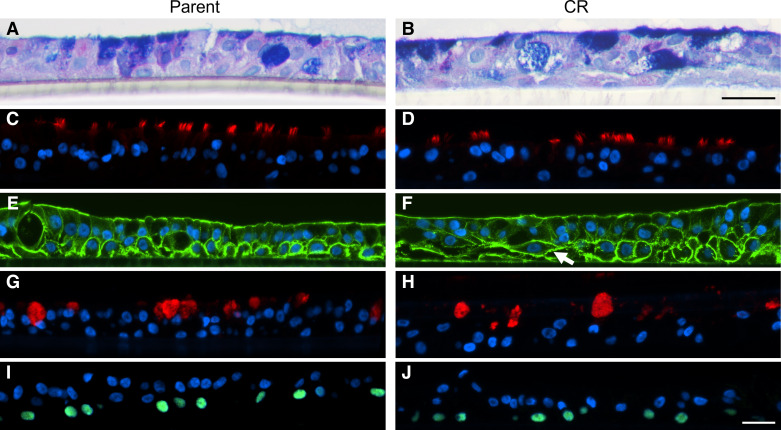
Cell morphology is similar between CR and parent pBECs differentiated at ALI culture except with an elongated cytoskeleton labeling in CR cultures. Representative images of parent and CR cells under light and fluorescent microscopy; AB/PAS labeling (*A* and *B*), cilia (anti-acetylated α-tubulin; *C* and *D*), cell cytoskeleton (anti-α-smooth muscle actin) showing elongated basal cell periphery (white arrow; *E* and *F*), mucus rich goblet cells (anti-MUC5AC; *G* and *H*), positive basal cells (anti-p63; *I* and *J*). Scale bar: 20 µm. AB/PAS, alcian blue/periodic acid Schiff; ALI, air-liquid interface; CR, conditional reprogramming; pBECs, primary bronchial epithelial cells.

The epithelial cell arrangement in differentiated CR cells generally resembled the parent pBECs at terminal differentiation (Fig. 1, *A*–*J*). No distinct differences in the presence, distribution, and morphology of total mucin glycoproteins were observed in AB/PAS-stained sections (Fig. 1, *A* and *B*). Fluorescent antibodies were used to label acetylated α-tubulin within cilia (Fig. 1, *C* and *D*), α-smooth muscle actin for cytoskeleton (Fig. 1, *E* and *F*), MUC5AC for mucus-secreting goblet cells (Fig. 1, *G* and *H*), and p63 for epithelial basal cells (Fig. 1, *I* and *J*). Fluorescent labeling was relatively consistent between parent and CR cells (Fig. 1, *C*–*J*), however, α-smooth muscle actin showed elongated structures in some areas superficial to basal cells (Fig. 1*F*; white arrow) in the CR group.

### Viral Replication Kinetics and Antiviral Responses Are Lower in CR Cells Compared with Parent pBECs

**Figure 2.**
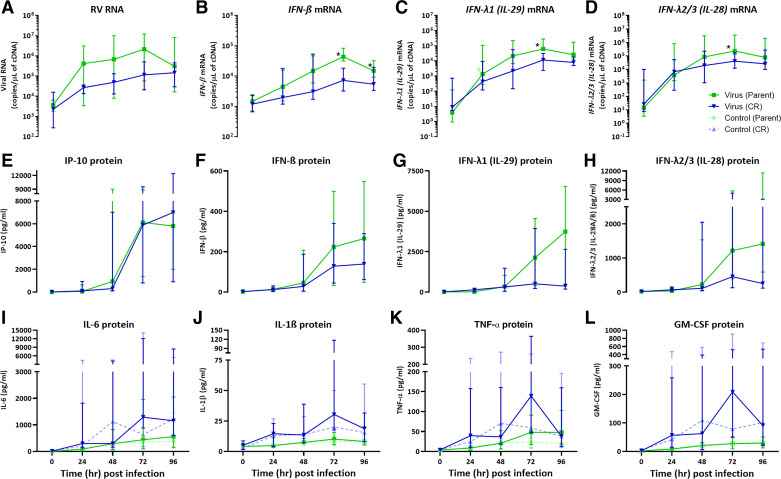
Viral replication and antiviral innate immune responses were reduced, but not proinflammatory cytokines from differentiated CR cells following RV-A1 infection in comparison with parent differentiated pBECs. Real-time quantitative PCR (RT-qPCR) was performed to quantify viral RNA (*A*) and mRNA for IFN-β (*B*), IFN-λ1 (IL-29; *C*), and IFN-λ2/3 (IL-28; *D*). A multiplex protein quantification assay (LegendPlex) was performed to determine changes in selected antiviral proteins from apical supernatant, IP-10 (*E*), IFN-β (*F*) and IFN-λ1 (*G*), IFN-λ2/3 (*H*) and cytokines, IL-6 (*I*), IL-1β (*J*), TNF-α (*K*), and GM-CSF (*L*) for 96 h following RV-A1 infection. Data presented as median ± IQR (*n* = 6). Following normality test, data were analyzed using a two-tailed Wilcoxon matched-pairs signed-rank test among the virus-infected groups at a time point for viral replication and antiviral responses. For multiple groups (proinflammatory cytokines), Kruskal–Wallis test with Dunn’s multiple corrections was used for each time point. *P < 0.05 was considered significant. CR, conditional reprogramming; GM-CSF, granulocyte-macrophage colony-stimulating factor; IQR, interquartile range; pBECs, primary bronchial epithelial cells; RV, rhinovirus.

There was an increase in viral RNA copy number following infection with RV-A1 MOI (0.001) in both parent and CR cells, however, with lower replication kinetics in CR cells (Fig. 2*A*). The peak viral RNA in parent cells was observed at 72 h postinfection (hpi), whereas for CR cells viral RNA increased consistently to 96 hpi. There was no significant difference in viral replication between the groups at any time point (Fig. 2*A*). Transcript copy numbers for *IFN-β, IFN-λ1*, and *IFN- λ2/3* were also induced following infection but were found to be significantly lower in the CR group at later time points (*P* = 0.0313*, IFN-β* at 72, 96 hpi, *IFN-λ1* and *IFN-λ2/3* at 72 hpi; Fig. 2, *B*–*D*). The peak IFN gene expression was observed at 72 hpi in both parent and CR groups (Fig. 2, *B*–*D*). IP-10, IFN-β, IFN-λ1, and IFN-λ2/3 proteins were induced at later time points following RV-A1 infection *(*Fig. 2, *E*–*H**).* Similar to gene expression, IFN protein responses were trended lower in cells expanded using the CR technique compared with the parent group between 48 and 96 hpi (Fig. 2, *F*–*H*), although these failed to reach significance. In contrast to IFN responses, IP-10 secretion was found to be trending higher at 96 hpi in the CR group following infection compared with the parent group (Fig. 2*E*).

Proinflammatory cytokines IL-6, IL-1β, TNF-α, and GM-CSF exhibited a trending increase in baseline expression over time in both CR and parent groups (Fig. 2, *I–L*). Although proinflammatory cytokine levels did not differ significantly between CR and parent cells, higher variability together with a trending increase of each measured cytokine was observed in CR group (Fig. *2*, *I–L*). Following RV-A1 infection, there was no significant induction of IL-6, IL-1β, TNF-α, or GM-CSF proteins in parent or CR cells relative to their noninfected controls (Fig. *2*, *I–L*).

### CR Cells Express Significantly Lower *FOXJ1* Gene Expression at Terminal Differentiation Compared with Parent Cells

**Figure 3.**
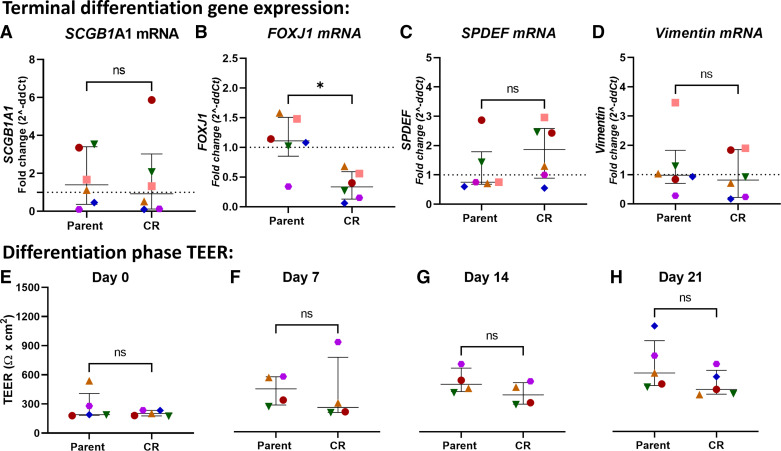
CR cells express significantly lower *FOXJ1* expression at terminal differentiation compared with parent cells. Terminal differentiation gene expression [fold change (2^ddct)] of SCGB1A1 (*A*), FOXJ1 (*B*), SPDEF (*C*) and Vimentin (*D*) were measured from CR cells and parent pBECs. Data presented as median ± IQR. Individual donors are represented with separate color-matched symbols, *n* = 6. Data were analyzed using a two-tailed Wilcoxon matched-pairs signed-rank test, *P < 0.05 was considered significant. Differentiation phase TEER measurements were recorded to noninvasively assess the barrier function of ALI cultures. Each TEER data point represents an average of 10 transwells from each donor during the differentiation phase. A color-matched symbol in both groups represents an individual donor. Sample size (*n* = 5) for parent and CR groups for *day 0* (*E*) and *day 21* (*H*); *n* = 4 for *day 7* (*F*) and *day 14* (*G*). Data were analyzed using a two-tailed Wilcoxon matched-pairs signed-rank test, *P < 0.05 considered significant. ALI, air-liquid interface; CR, conditional reprogramming; IQR, interquartile range; TEER, transepithelial electrical resistance.

Following terminal differentiation, CR cells showed significantly lower *FOXJ1* gene expression (marker of ciliogenesis) when compared with parent pBECs, *P* = 0.0313 (Fig. 3*B*) without any changes in other phenotypic markers, *SCGB1A1* (club cells), *SPDEF* (goblet cells), and *Vimentin* (mesenchymal cells; Fig. 3, *A*, *C*, and *D*). The TEER measurements for each donor were not significantly different between the parent and CR cultures from the day of ALI establishment to the day of experimentation i.e., *day 0* to *day 21* (Fig. 3, *E*–*H*).

## DISCUSSION

For the first time, we have directly compared pBECs obtained from donors with asthma with their respective CR cells at terminal differentiation; assessing morphology, baseline gene expression, barrier function, and interferon production following RV-A1 infection. We have shown that CR cells differentiate at ALI culture and closely resemble their parent cells with an exception of cell cytoskeleton elongation superficial to basal cells. We also found that CR cultures exhibited no difference in barrier function compared with their parent cell cultures. In addition, these CR cells from donors with asthma express significantly lower *FOXJ1* in comparison with “parent” pBECs at terminal differentiation. Finally, IFN mRNA was significantly lower in CR cultures, whereas viral replication trended lower over time compared with parent cultures, following RV-A1 infection.

The pBECs obtained from individuals with or without respiratory diseases such as asthma have a limited proliferative capacity (21). These cells differentiate at ALI culture, to closely mimic the phenotype of the hosts’ airway epithelium (22). To differentiate these cells at ALI culture incurs cost and due to limited proliferative capacity restricts a variety of experimental conditions achievable per donor. Earlier, there have been numerous approaches to overcome limited proliferation ability including immortalization through genetic modifications such as overexpression of retrovirus-mediated human telomerase reverse transcriptase (*hTERT*), cyclin-dependent kinase 4 (*Cdk-4*), and viral oncogenes (23–25). Although these genetically modified cells (cell lines) proliferate over extended passages, mostly they fail to properly differentiate at ALI culture (26). Recently, the CR technique was developed to proliferate pBECs for extended passages using a combination of ROCK inhibitor (Y-27632) and γ-irradiated 3T3 fibroblasts, a nongenetic modification (2). Following the removal of ROCK inhibitor, CR-pBECs differentiate at ALI culture (2, 4, 5). Although these cells differentiate at ALI culture and appear similar to “parent” pBECs, there are reports which demonstrate a loss of functional characteristics and epithelial cell composition from extended passage cells from cystic fibrosis donors and restored with a modification of CR methodology (3, 6). Using the same CR technique, cells obtained from donors with asthma are also expanded at multiple passages and differentiated at ALI culture from passage one CR (4). In addition, there are no reports directly comparing differentiation status and antiviral responses from CR cells with their respective parent donors with asthma pBECs, hence the current study.

Using the published protocols (2, 4, 5), we were successful in expanding CR asthma cells in monolayer cultures. But, upon raising to ALI culture using BEGM media (13), they did not establish to ALI. We, therefore, weaned the ROCK inhibitor as described in the materials and methods. This minor adjustment to the CR expansion medium restored the cells’ ability to differentiate at ALI with no difference in barrier function during the differentiation phase. Following differentiation, cells were labeled with fluorescent antibodies for specific protein markers localization (17). We found that the CR cell’s cytoskeleton appeared elongated with no difference in cell membrane height nor any immediate difference in the appearance of goblet cells or cilia from cross-sectional images. Previously, ROCK inhibitor has been shown to alter the biochemical properties of a cell by influencing the reorganization of cytoskeletal microfilaments and microtubules in retinal pigment epithelium (27). Future detailed studies of the cell cytoskeleton are warranted in CR cells before their use to answer research questions where cytoskeletal arrangements may be critical.

We demonstrated a reduction in *FOXJ1* gene expression in CR cells compared with parent asthma pBECs at terminal differentiation. *FOXJ1* is a transcription factor and a key regulator involved in the differentiation of motile ciliated cells from progenitor cells (28, 29). Lower *FOXJ1* expression results in the loss of apical docking of ezrin (protein required for linking apical cytoskeleton and plasma membrane) and basal bodies (modified centrioles that give rise to cilia or flagella) with the ultimate loss of axoneme structure (30–32). Fewer ciliated cells reduce the efficiency of mucus clearance in the cell culture environment and may impact specific disease models, such as asthma, chronic obstructive pulmonary disease (COPD), and also their associated exacerbation models including infections where numbers of ciliated cells are critical. Restoring *FOXJ1* expression in CR cells to mimic parent donor cells may be possible but calls into question the equivalence of CR to parent cells more generally.

Immediately following a 6-h RV-A1 incubation, we found no difference in viral RNA copy number in both CR and parent cells, indicating a similar viral entry into the cells. However, there was a trending lower viral replication in CR cells from 24 to 96 hpi compared with parent cells. In contrast, IFN gene expression was significantly lower from differentiated CR cells compared with parent cells at 72 and 96 hpi. These viral replication kinetics and antiviral responses may potentially link to lower *FOXJ1* expression in CR cells. Although RV-A1 has been shown to have preferential binding to the basal cells of the epithelium (33), the core binding receptor low-density lipoprotein receptor (LDL-R) is also found on ciliated cells of the epithelium although in less abundance (34). We anticipate that lower FOXJ1 gene expression influences the differentiation of ciliated cells in CR cultures and thus leads to a lower replication capacity for RV, blunting the viral replication curve. Based on these data, a cautious approach should be taken when choosing CR cells for experiments requiring binding to or assessment of multiciliated cells. For example, RV-C, a separate strain of RV identified first in 2006 (35, 36) uses CDHR3 on ciliated cells for virus entry (37). In addition, it has been reported that *FOXJ1* expression reduces inflammatory signaling via NF-κB transcription regulator (38), and as such, reduction in *FOXJ1* through conditional reprogramming may bias inflammatory and antiviral responses. In this study, we found no significant difference in inflammatory responses from CR cells in comparison with parent asthma pBECs.

The aberrant *FOXJ1* response from differentiated CR cells may be due to the use of irradiated 3T3 cells and ROCK inhibitor during the expansion phase, which are essential for immortalization of cells (2, 5, 39). There is no literature on how these conditions influence the differentiation of epithelial cells after removal in comparison with parent cells or cause any epigenetic changes in the cells during the expansion phase. Future studies are warranted to identify the possible role of irradiated 3T3 cells and ROCK inhibitor in proper differentiation of CR cells at ALI cultures.

Although this study is the first to provide an in-depth comparison of donor-specific parent and CR cells, it is also limited to low sample numbers and FOXJ1 protein assessment. The number of available transwell cultures of primary cells was also limited and therefore we were limited to using just one minor group RV strain. Increasing the number of donor-derived samples in future studies may render proinflammatory cytokine and RV replication data as statistically significant and offer further insights into changes associated with the use of CR technique. In addition, using multiple RV strains with diverse viral entry mechanisms could provide more information on entry/replication kinetics and antiviral responses.

In summary, the CR technique is beneficial in proliferating cells over extended passages without any gross genetic modifications. The unique advantage of CR cells over other cell lines is the ability to differentiate these cells at ALI culture and mimic the airway epithelium. As CR techniques vary between research groups, it is essential that the most appropriate technique is utilized to best match individual cell function and morphology to the scientific question being posed. It is important to recognize that the CR technique is a tool that enables the prolonged use of a finite resource in vitro and as such does not perfectly reproduce the in vivo airway epithelium upon ALI differentiation. By recognizing the limitations of such a technique and modeling experiments with this in mind the CR technique will remain a critical tool for the study of airway epithelial cells from all backgrounds.

## DATA AVAILABILITY

Data will be made available upon reasonable request.

## GRANTS

This work was funded by the Australian National Health and Medical Research Council (NHMRC—https://www.nhmrc.gov.au/; APP1145332).

## DISCLOSURES

Nathan Bartlett is an editor of American Journal of Physiology-Lung Cellular and Molecular Physiology and was not involved and did not have access to information regarding the peer-review process or final disposition of this article. An alternate editor oversaw the peer-review and decision-making process for this article. None of the other authors has any conflicts of interest, financial or otherwise, to disclose.

## AUTHOR CONTRIBUTIONS

P.C.V., N.W.B., D.A.K., C.L.G., and A.T.R. conceived and designed research; P.C.V., K.S.N., J.M.R., and A.T.R. performed experiments; P.C.V. and A.T.R. analyzed data; P.C.V., N.W.B., P.A.B.W., D.A.K., C.L.G., and A.T.R. interpreted results of experiments; P.C.V. and A.T.R. prepared figures; P.C.V. drafted manuscript; P.C.V., C.L.G., and A.T.R. edited and revised manuscript; P.V., K.S.N., N.W.B., P.A.W., D.A.K., C.L.G., and A.T.R. approved final version of manuscript.
